# Local spatial analysis: an easy-to-use adaptive spatial EEG filter

**DOI:** 10.1152/jn.00560.2019

**Published:** 2020-11-11

**Authors:** R. J. Bufacchi, C. Magri, G. Novembre, G. D. Iannetti

**Affiliations:** ^1^Neuroscience and Behaviour Laboratory, Istituto Italiano di Tecnologia, Rome, Italy; ^2^Department of Neuroscience, Physiology and Pharmacology, University College London, London, United Kingdom

**Keywords:** EEG components, electroencephalography (EEG), event-related potentials (ERPs), referencing, spatial filtering

## Abstract

Spatial EEG filters are widely used to isolate event-related potential (ERP) components. The most commonly used spatial filters (e.g., the average reference and the surface Laplacian) are “stationary.” Stationary filters are conceptually simple, easy to use, and fast to compute, but all assume that the EEG signal does not change across sensors and time. Given that ERPs are intrinsically nonstationary, applying stationary filters can lead to misinterpretations of the measured neural activity. In contrast, “adaptive” spatial filters (e.g., independent component analysis, ICA; and principal component analysis, PCA) infer their weights directly from the spatial properties of the data. They are, thus, not affected by the shortcomings of stationary filters. The issue with adaptive filters is that understanding how they work and how to interpret their output require advanced statistical and physiological knowledge. Here, we describe a novel, easy-to-use, and conceptually simple adaptive filter (local spatial analysis, LSA) for highlighting local components masked by large widespread activity. This approach exploits the statistical information stored in the trial-by-trial variability of stimulus-evoked neural activity to estimate the spatial filter parameters adaptively at each time point. Using both simulated data and real ERPs elicited by stimuli of four different sensory modalities (audition, vision, touch, and pain), we show that this method outperforms widely used stationary filters and allows to identify novel ERP components masked by large widespread activity. Implementation of the LSA filter in MATLAB is freely available to download.

**NEW & NOTEWORTHY** EEG spatial filtering is important for exploring brain function. Two classes of filters are commonly used: stationary and adaptive. Stationary filters are simple to use but wrongly assume that stimulus-evoked EEG responses (ERPs) are stationary. Adaptive filters do not make this assumption but require solid statistical and physiological knowledge. Bridging this gap, we present local spatial analysis (LSA), an adaptive, yet computationally simple, spatial filter based on linear regression that separates local and widespread brain activity (https://www.iannettilab.net/lsa.html or https://github.com/rorybufacchi/LSA-filter).

## Graphical abstract

**Figure:**
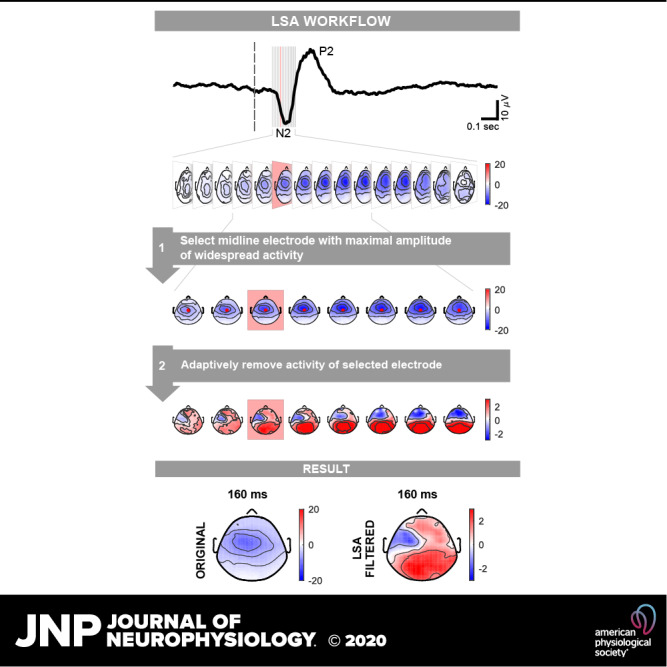

## INTRODUCTION

Event-related potential (ERP) experiments are conducted to investigate how the central nervous system processes sensory, motor, or cognitive events. ERPs comprise several components that often reflect the activity of distinct cortical generators (1). Because these components overlap in time and space, isolating them requires spatial filtering, a mathematical procedure that changes the EEG voltage value at each electrode according to a weighted combination of voltage values at two or more other electrodes. There are two types of spatial filters, “stationary” and “adaptive.”

**Figure 1.**
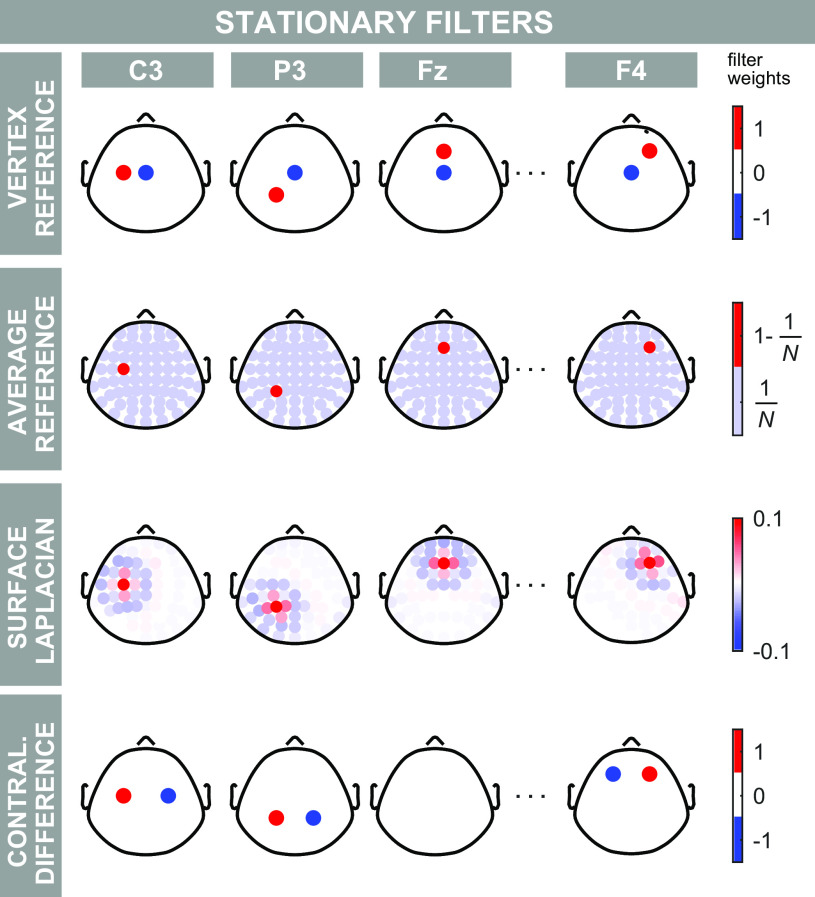
Overview of stationary filters. Stationary filters use weights defined a priori. Each row shows these weights for a type of stationary filter. Each column shows the weights applied to a number of representative electrodes. Nonzero weights are plotted in color. When the filters are applied to the data, these weights are multiplied by the voltage at each channel. The resulting weighted voltage values constitute the filter outputs. In the vertex reference (first row) the signal at Cz is subtracted from the signal of each given electrode. Therefore, for each electrode, the VR filter has two non-zero weights: the weight of the electrode of interest is 1, and the weight of electrode Cz is −1. In the average reference (second row) the average signal across all *N* electrodes is subtracted from each given electrode. Therefore, 1 − 1/*N* is the weight for the electrode of interest and 1/*N* is the weight for all other electrodes. In the surface Laplacian (third row) a weighted local combination of signal is subtracted from each given electrode. Finally, the contralateral difference (bottom row) computes the signal difference between each given electrode and its symmetrical electrode with respect to the sagittal midline. Color axes represent electrode weights.

Stationary spatial filters do not change across time and space because their weights are defined a priori (Fig. 1). The most widely used stationary spatial filters are the vertex reference (VR; subtracting from each electrode the activity of Cz) (2–8), the average reference (AR; subtracting the average of all electrodes) (1, 9, 10), the surface Laplacian (SL; second-order spatial derivatives) (10, 11), and the contralateral difference (CD; subtracting from a given electrode the activity of its symmetrical electrode with respect to the sagittal midline, e.g., C3 minus C4) (10, 11). The popularity of these methods stems from their immediacy: they are conceptually simple, easy to use, and fast to compute. The problem is that they all assume that the EEG signal is stationary, i.e., that it does not change across sensors and time. Nonstationarities, however, are the essence of ERP recordings. They are exactly the troughs and peaks observed in the time course and the scalp maps of the EEG responses. Several groups have shown that applying stationary spatial filters to EEG nonstationarities can lead to misinterpretations of the measured activity (1, 6, 12–14).

Adaptive spatial filters, in contrast, infer their weights directly from the spatial properties of the data, and thus do not present the shortcomings of stationary filters (15). Commonly used adaptive filters are principal component analysis (PCA; extracting the components that explain most of the variance in the signal) (16, 17), independent component analysis (ICA; extracting components that are statistically independent) (18–21), as well as source localization techniques, such as beamforming (identifying likely brain sources) (15, 22). The issue with these adaptive filters is that using them is not trivial. Understanding how they work and how to set the necessary parameters requires advanced statistical and physiological knowledge. These filters estimate many components or sources that must be sorted, categorized, or matched to anatomical models. In addition, they are computationally intensive. Thus, researchers often refrain from using the adaptive techniques for extracting physiologically relevant neural components and settle for the simpler but less effective stationary alternatives.

**Figure 2.**
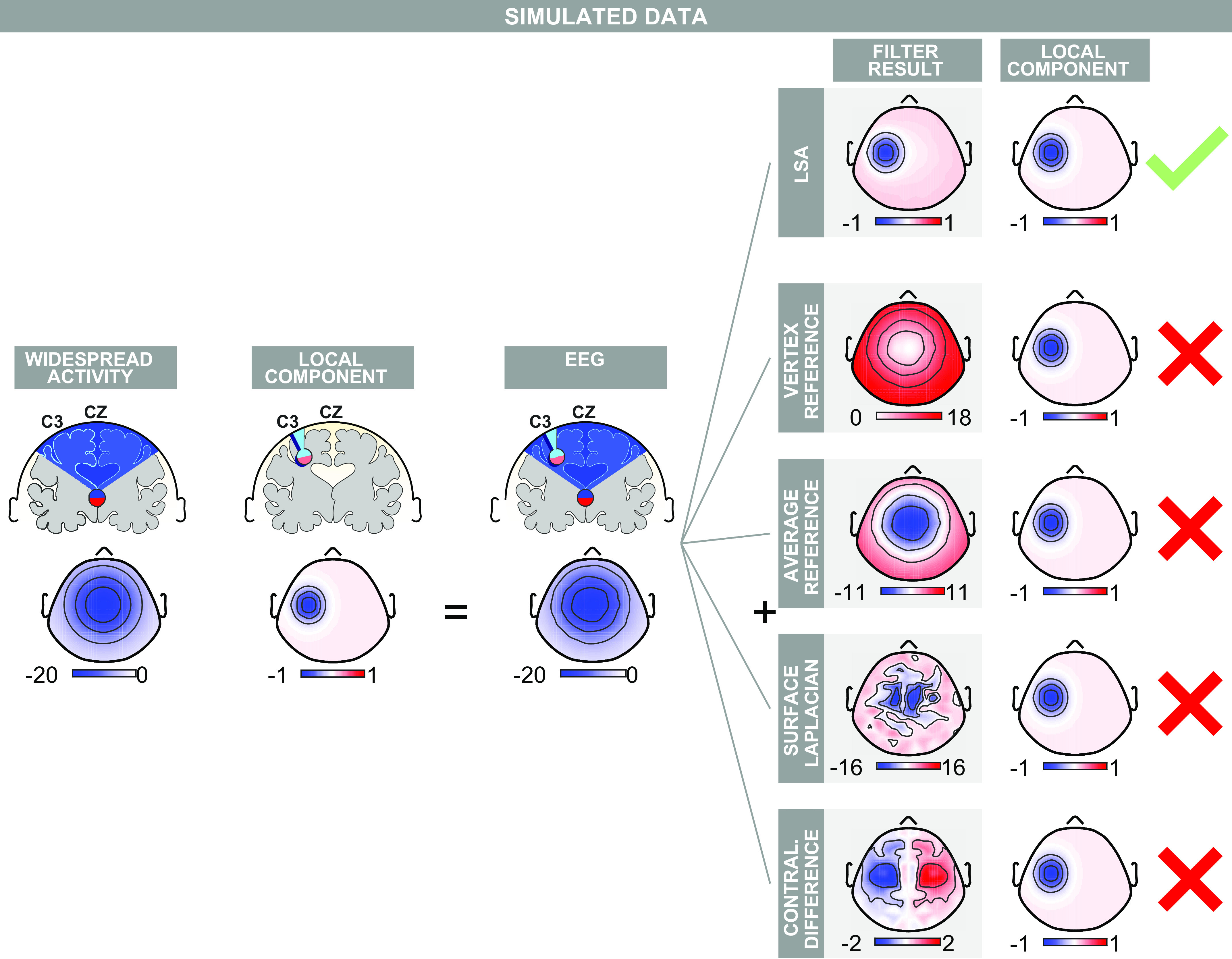
Performance of local spatial analysis (LSA) on simulated event-related potential (ERP) data containing a central widespread component and a lateralized local component. *Left*: EEG topographies were generated as a widespread component at Cz plus a local negative component at C3. *Right*: by exploiting the information contained in the trial-by-trial variability of the simulated data, LSA returns a scalp topography virtually identical to that of the original local component. Commonly used stationary filters (vertex reference, average reference, surface Laplacian) failed to highlight the local component. Only the contralateral difference returned a negativity in the left hemisphere, although it also created a spurious positivity in the contralateral hemisphere. Color axes represent voltage (µV).

In this work, we propose a new adaptive filter specifically designed for highlighting local ERP components masked by large widespread activity. Despite being adaptive both in time and space, this approach is easy to use and conceptually simple. The core idea is to exploit the information available in the trial-by-trial variability of stimulus-evoked neural activity. Far from being noise, this variability is largely due to the activity generated by the brain itself (23–25) and contains precious information about the brain activity that results in the EEG signal at different sites (24, 26). The filter that we propose specifically exploits these trial-by-trial fluctuations when there is a widespread EEG source captured at multiples sites (Fig. 2). This source produces trial-by-trial fluctuations that are necessarily correlated at the two sites, as the propagation of an electrical field is virtually instantaneous (10). Widespread sources are not the only cause of correlated variability. Two local sources can also produce correlated trial-by-trial fluctuations at faraway sites, for example, when two cortical sources are driven by the activity of a third subcortical structure. In this case, however, we also expect to observe uncorrelated trial-by-trial fluctuations reflecting, among others, nonshared input, intrinsic variability of each generator, or the differential effect of neuromodulators on different generators.

These considerations suggest that extracting at each electrode the part of EEG signal that is statistically independent to the widespread activity could highlight local ERP components, despite the presence of a concomitant widespread activity. We propose that this goal can be achieved with a novel approach based on simple linear regression, and that we call local spatial analysis (LSA). LSA is not designed to filter the entire ERP waveform. Instead, it should be used on the time window of the ERP waveform within which a widespread scalp potential is present. The influence of that widespread activity is then linearly regressed out of the signal, across trials, at all recording sites. The resulting filtered EEG signal will consequently show local activities that were previously masked by the widespread potentials, and that have different trial-by-trial variability to the widespread potential. We use both simulated and real ERP data elicited by auditory, nociceptive somatosensory, nonnociceptive somatosensory, and visual stimuli, to show how LSA outperforms widely used stationary filters in identifying local ERP components.

## METHODS

### Stationary Spatial Filters

Figure 1 shows a summary of the commonly used stationary spatial filters.

#### Vertex reference.

The vertex reference (VR) subtracts the EEG signal at Cz from the activity at each electrode (6). The output of the filter at Cz is therefore zero (Fig. 1, first row).

#### Average reference.

The average reference (AR) subtracts the average activity of all EEG electrodes from the activity of each electrode (10) (Fig. 1, second row).

#### Surface Laplacian.

The surface Laplacian (SL) is a second-order spatial derivative. Several approximations for the SL have been developed for EEG analysis (11), and research on the topic is ongoing (27). These approximations replace the voltage at each electrode with a weighted sum of a combination of the voltage at the electrode of interest and its neighboring electrodes (Fig. 1, third row; this operation can be imagined as spatially convolving a basis function given by the weights with the EEG data). SL has several appealing theoretical qualities, such as reducing volume conduction effects, and thus attenuating widespread activities (10). Here, we applied the method described by Perrin et al. (28), with smoothing factor equal to 10^−5^, order of the Legendre polynomials equal to 80, and spherical spline order equal to 3. These are standard parameters for performing a surface Laplacian with the EEG setup used in this study (29).

#### Contralateral difference.

The contralateral difference (CD) subtracts from the EEG signal of each electrode the signal of the electrode symmetrical with respect to the sagittal midline (e.g., C3 minus C4, or P7 minus P6) (1, 30). The voltage at all electrodes on the sagittal midline is, therefore, zero (Fig. 1, fourth row).

### Adaptive Spatial Filters

#### Independent component analysis.

Independent component analysis (ICA) attempts to decompose the EEG signal into statistically independent components (ICs). Several implementations of this approach exist (31). We applied the extended-ICA method implemented in EEGLAB (32, 33).

#### Principal component analysis.

Principal component analysis (PCA) attempts to extract the components that explain most of the signal variance (16, 17). We used the singular value decomposition approach implemented in the standard MATLAB statistics toolbox.

### Local Spatial Analysis

Given the EEG signals *S*_A_ and *S*_B_, measured at two electrodes A and B at a given time point, LSA uses a simple linear regression of *S*_A_ on *S*_B_ to highlight the local activity at A. For the results of LSA to be interpretable, some assumptions must hold. First, we assume that we can write the signals at *S*_A_ and *S*_B_ as the sums of widespread and local contributions:

$$S_{B}=W_{B}+L_{B}$$

and

$$S_{A}=\lambda \cdot W_{B}+L_{A}.$$

*W*_B_ denotes the part of the signal measured at electrode B, which is produced by the source of a widespread component. We assume that the amplitude and trial-by-trial variation of this component are maximal at electrode B. Therefore, −1 < λ < 1 is a scaling factor that reflects the assumption that the widespread field is smaller at electrode A than at electrode B. *L*_A_ and *L*_B_ are the local components at A and B, and thus do not respectively contribute to S_B_ and S_A_.

We denote with cov{·;·} and var{·} the covariance and variance operators, respectively. The larger var{*W*_*B*_} is, the closer the following approximation becomes:

$$\lambda \approx \frac{cov\left\{S_{A};S_{B}\right\}}{\text{var}\left\{S_{B}\right\}}=\lambda^{\prime }.$$

Thus, if the widespread component is large enough,

$$S_{A}-\lambda^{\prime }\cdot S_{B}\approx S_{A}-\lambda \cdot S_{B}=L_{A}-\lambda \cdot L_{B.}$$

Then, because |λ|<1, and assuming *L*_B_ is small, we have that

$$S_{A}-\lambda^{\prime }\cdot S_{B}\approx L_{A}.$$

In other words, LSA can regress widespread components out of the signal because of their trial-by-trial variability. This reveals local activities. However, if the widespread activity does not vary from trial to trial, or is not present in the first place, there is nothing to regress out.

**Figure 3.**
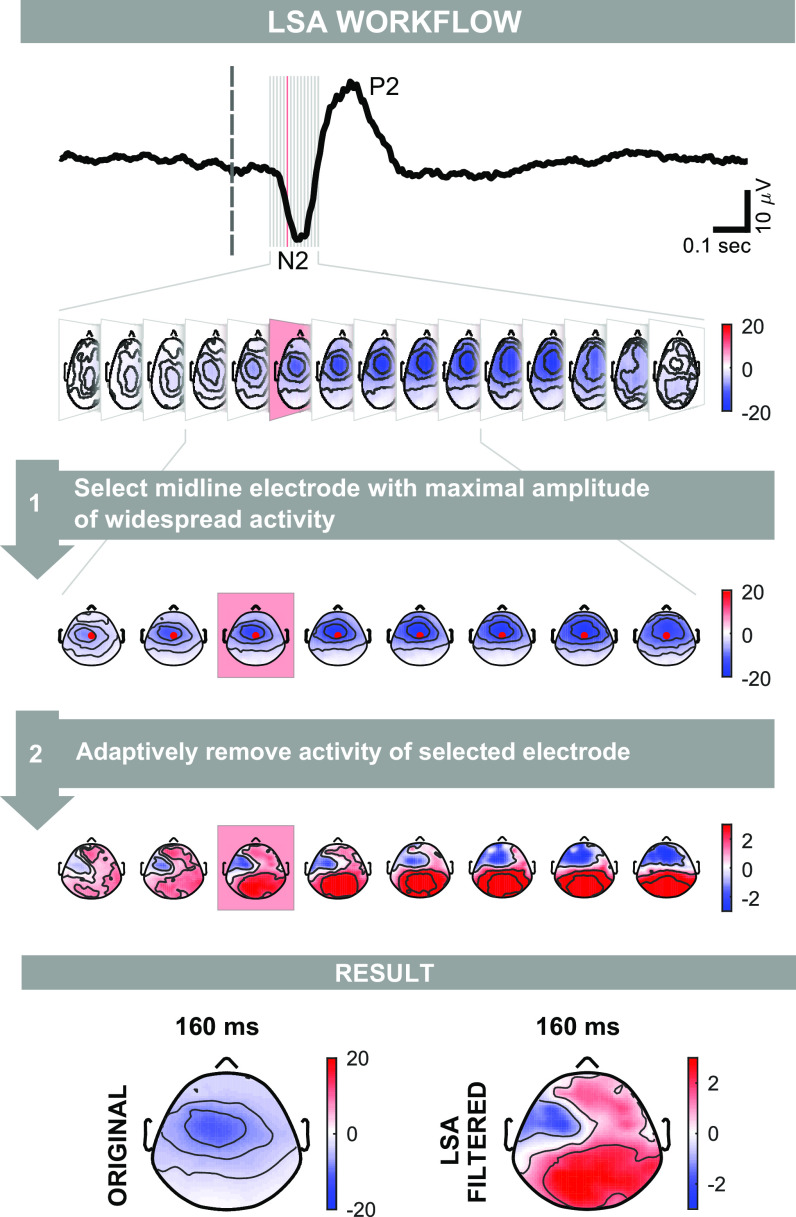
Procedure for filtering EEG data using local spatial analysis (LSA). The filter is applied to real laser-evoked event-related potentials (ERPs). *Step 1*: first, the electrode at which the widespread N2 component is maximal in amplitude is identified in the grand average scalp map series. In this example, the electrode is Cz. Second, the time interval including the widespread signal (i.e., the interval during which most of the electrodes have the same polarity) is selected. In this example, the interval is 130–220 ms. *Step 2*: LSA exploits the different trial-by-trial variability of the local vs. the widespread component, and thereby removes the activity from the identified electrode (Cz) from all other electrodes. This is done separately for each subject and time point. The last row shows the comparison of the topography at the same time point (in this example, 160 ms) before and after filtering with LSA. Color axes represent voltage (µV).

Figure 3 illustrates how LSA works on real EEG data. First, we identify, in the group-average response, the time intervals with a large widespread scalp activity (in the example shown in Fig. 3, a negativity). We then select the electrode El_max_ at which this widespread activity has maximal amplitude (in the example shown in Fig. 3, electrode Cz). The further implicit assumption is that components with large amplitude will also have large trial-by-trial variability. Subsequently, we linearly regress out the signal at El_max_ from each other electrode. It is important to note that the estimation of λ and the linear regression are performed separately for each time point, electrode, and subject. Thus, the proposed filter is adaptive both in time and space. The estimation is performed using the statistical information available in the distribution of the trials recorded from a single subject. We suggest that at least 20–30 trials per subject are necessary for the estimation to be robust, and all trials should be collected under the same stimulation condition. LSA is theoretically simple, easy to use, and can be freely downloaded from https://www.iannettilab.net/lsa.html or https://github.com/rorybufacchi/LSA-filter. For a tutorial on how to set up the plugin, see https://www.youtube.com/watch?v=-Il3Qhfurnk.

The mathematical simplicity of LSA leads to very short computational times: when performed on real data from one participant, the call to the LSA function took 8.3 ± 4.5 ms to run, whereas AR took 8.1 ± 4.5 ms to run (100 repetitions, using a machine with Intel(R) Core i7-8750H CPU @ 2.20 GHz, 32GB RAM).

### Simulated EEG Data

We simulated EEG data sampled from 120 electrodes, according to the International 10-5 system, as follows. At each electrode the EEG signal was the sum of a widespread and of one or more local fields (Fig. 2). The widespread field was modeled as an inverted 2-D Gaussian with a negative peak of −20 µV at Cz and a standard deviation of half a cap radius (the cap radius is ∼14.7 cm, computed as the maximal distance between any electrode and Cz). The local field had a negative peak of −1 µV at either Cz or Fz, depending on the case considered, and a standard deviation of 20% of the cap radius. When we added two additional local fields, their peaks were of +1 µV and −0.7 µV, and standard deviations of 20% and 30% of the cap radius, respectively.

We included three types of variability in the simulated responses. The first type of variability was changes in the amplitude of the widespread and the local sources. This variability was modeled using multiplicative random noise (Gaussian distribution, mean = 1, standard deviation = 1). This noise was added independently to the widespread and to the local components and to each trial. The second type of variability was overall electrical or neural variability. This variability was modeled as additive noise (Gaussian distribution, mean = 0 µV, standard deviation = 1 µV). This noise was added identically to each electrode, separately for each trial. The third type of variability was differences in electrode conductance. This variability was modeled as multiplicative noise (Gaussian distribution, mean = 1, standard deviation = 0.05). This variability was added separately to each electrode but identically for each trial. Forty trials were generated. To test the robustness of the filter to higher levels of noise, we ran four additional simulations, where we multiplied all sources of nonneural noise by factors of 2, 4, 6, and 8.

### Recorded EEG Data

Recorded EEG data were reported in Mouraux and Iannetti (21) and Liang et al. (19), where detailed information about the data collection and experimental paradigm can be found. Here, we only provide a short summary of research participants, employed stimuli, experimental procedure, and EEG recording.

#### Participants.

Nineteen healthy volunteers (2 females; aged 25 ± 6 years; 1 left-handed) took part in the studies. Before the electrophysiological recording, participants were familiarized with the experimental setup. They were also exposed to a small number of test stimuli (5–10 stimuli for each stimulus type). All experimental procedures were approved by the local Ethics Committee. Written informed consent was obtained from all participants.

#### Experimental procedure.

The experiment took place in a dim, quiet, and temperature-controlled room. Participants were seated in a comfortable armchair placed in front of a desk. They were told to relax, minimize eye blinks, and keep their gaze fixed on a white cross (3 × 3 cm) placed centrally in front of them, at an eye distance of ∼40 cm. Brief stimuli of different sensory modalities (auditory, nociceptive somatosensory, nonnociceptive somatosensory, and visual) were intermixed and randomly delivered to or near the dorsum of the right hand to ensure that differences in the recorded responses were not related to differences in spatial attention. In Liang et al. (19), stimuli were also separately delivered to or near the dorsum of the left hand. Furthermore, in Liang et al. (19), somatosensory stimuli were only nonnociceptive. Only one stimulus belonging to one stimulus modality was presented on each trial. Stimuli were presented in four successive blocks. The interstimulus interval varied randomly between 5 s and 10 s (rectangular distribution). Each block was separated by a 3- to 5-min break. To ensure that vigilance was maintained throughout the experiment, and that each type of sensory stimulus was equally relevant to the task, participants were instructed to report the total number of perceived stimuli at the end of each of the four blocks.

#### Sensory stimuli.

The hands of the participants were placed at an eye distance of ∼45 cm, 25° left or right from the midline, 30° below eye level. Nociceptive somatosensory stimuli were heat laser pulses [Nd:YAP, 4 ms duration (34)] delivered on the innervation territory of the right superficial radial nerve. Nonnociceptive somatosensory stimuli were constant current square-wave electrical pulses (1 ms duration; DS7A, Digitimer Ltd, UK) delivered through a pair of skin electrodes (1 cm interelectrode distance) placed on the wrist, over the median nerve. Visual stimuli were brief flashes (50 ms duration) delivered through green light-emitting diodes (11.6 cd, 15° viewing angle) mounted on the top of the speaker. Auditory stimuli were brief 800-Hz tones (50-ms duration; 5-ms rise and fall times) presented at a loud but comfortable listening level (85 dB SPL) and delivered through a speaker (VE100AO, Audax) placed in front of the participant’s hand. Stimulus saliency was similarly rated across modalities. Further information can be found in Mouraux and Iannetti (21) and Liang et al. (19).

#### EEG recordings.

The EEG was recorded using 124 electrodes placed on the scalp according to the International 10-5 system, using the nose as reference. Ocular movements and eye blinks were recorded using two surface electrodes, one placed over the right lower eyelid, the other placed ∼1 cm lateral to the lateral corner of the right orbit. The electrocardiogram was recorded using two surface electrodes placed on the volar surface of the left and right forearms. Signals were amplified and digitized using a sampling rate of 1024 Hz (SD128, Micromed, Italy). Electrodes FFT9H, F7, FFT10H, and F8 were removed from the analysis because the recorded signal was extremely noisy. For the remaining 120 electrodes, signal preprocessing was conducted using MATLAB (MathWorks). EEG signals were segmented into separate 4-s-long epochs (−2 s to +2 s relative to stimulus onset). Line noise was removed using the procedure described in Eschenko et al. (35). Epochs were band-pass filtered (1 to 100 Hz; 3rd order forward and reverse direction Butterworth filter). All epochs were visually inspected, and trials associated with large muscular artifacts were removed from the analysis (∼1 trial per subject and condition was removed, i.e., ∼2% of all trials). Faulty or noisy electrodes were interpolated by replacing their signal with the average of the surrounding ones (∼1 electrode per subject). Artifacts due to blinks or eye movements were corrected using ICA (36). All aforementioned steps were repeated separately for each stimulus modality. Following this offline processing, a mean of 44 trials (range 35–61 trials) was available for each subject and each modality. Note that this is a simple procedure to clean the data, which could be easily automated. This is important, as it highlights the ease of generating data that can be fed into LSA. Finally, we assessed the difference from zero at the minimum of each negative local potential identified by the filters using one-tailed *t* tests.

## RESULTS

### Simulations

We tested whether the novel adaptive filter, local spatial analysis (LSA), could successfully retrieve local components masked by strong widespread activity in simulated data. To this aim, we simulated 40 trials of one time point of an ERP response in which a weak and local negative component centered at C3 overlapped with a strong and widespread negative component centered at Cz (Fig. 2; see also methods). The generated EEG had an average amplitude of −20 ± 3 µV at Cz (means ± standard error). The trial-by-trial variability was comparable if not higher than in recorded ERP data (see next section). Nonetheless, we additionally performed identical analyses to those described in the remainder of this section, but with substantially higher levels of noise. The results, shown in Supplemental Fig. S1 (all Supplemental Material available at https://doi.org/10.6084/m9.figshare.12104043), were qualitatively similar to those described here, as long as the noise was not extreme (i.e., standard deviations of global noise >5 times larger than the local component mean amplitude and variance). Note that the weak local component was barely noticeable in the average scalp EEG topography (Fig. 2).

The widespread component produced a positive trial-by-trial correlation between C3 and Cz (not shown). We estimated the linear regression coefficient of C3 on Cz. This coefficient quantifies the degree by which one must subtract the EEG signal at Cz from that at C3 to remove the common widespread effect. By repeating this weighted subtraction against Cz for all electrodes, LSA returned a scalp topography virtually identical to that of the simulated local component (Fig. 2).

We also applied four of the most commonly used stationary spatial filters to the same data (Fig. 2; see also methods). First, we attempted to remove the widespread component by mere subtraction of the potential at Cz using the vertex reference (VR). VR failed to highlight any local activity: at peripheral electrodes VR leaked signal from the vertex and returned spurious positive EEG signal. A similar result was obtained with the average reference (AR), although in this case the peripheral leakage was less pronounced. The surface Laplacian (SL) returned scalp maps that were spatially noisy, and in which it was hard to identify the lateralized component. The contralateral difference (CD) returned a localized negative component. The shape of the lateralized negativity returned by CD, however, was not identical to that of the generated local activity. Furthermore, CD also created a spurious symmetrical positive component. We also observed two additional problems with this approach. First, CD returned no signal when we moved the local component from C3 to a site on the medio-lateral axis (e.g., at Fz, Supplemental Fig. S2). Second, unlike LSA, CD returned a local negativity at C3 when we simulated that the EEG cap was slightly misplaced (3-mm shift to the right) along the medio-lateral axis, even though in this case no local activity was present in the simulated data (Supplemental Fig. S2).

We also applied ICA and PCA to the same data (see methods). For ICA, the small number of samples available (40 trials, 1 time point) heavily constrained the number of independent components that could be extracted from the data. Thus, we extracted four independent components (ICs) without incurring in numerical errors. The widespread activity dominated all extracted ICs (Supplemental Fig. S3). Although there was a hint of lateralization in IC3, none of the ICs clearly highlighted the small local activity. For PCA, we used the same number of components as for ICA. PCA split the simulated activity into widespread, local, and channel noise remarkably well, when there was only a single local component (Fig. S4, Supplemental Fig. S5).

**Figure 4.**
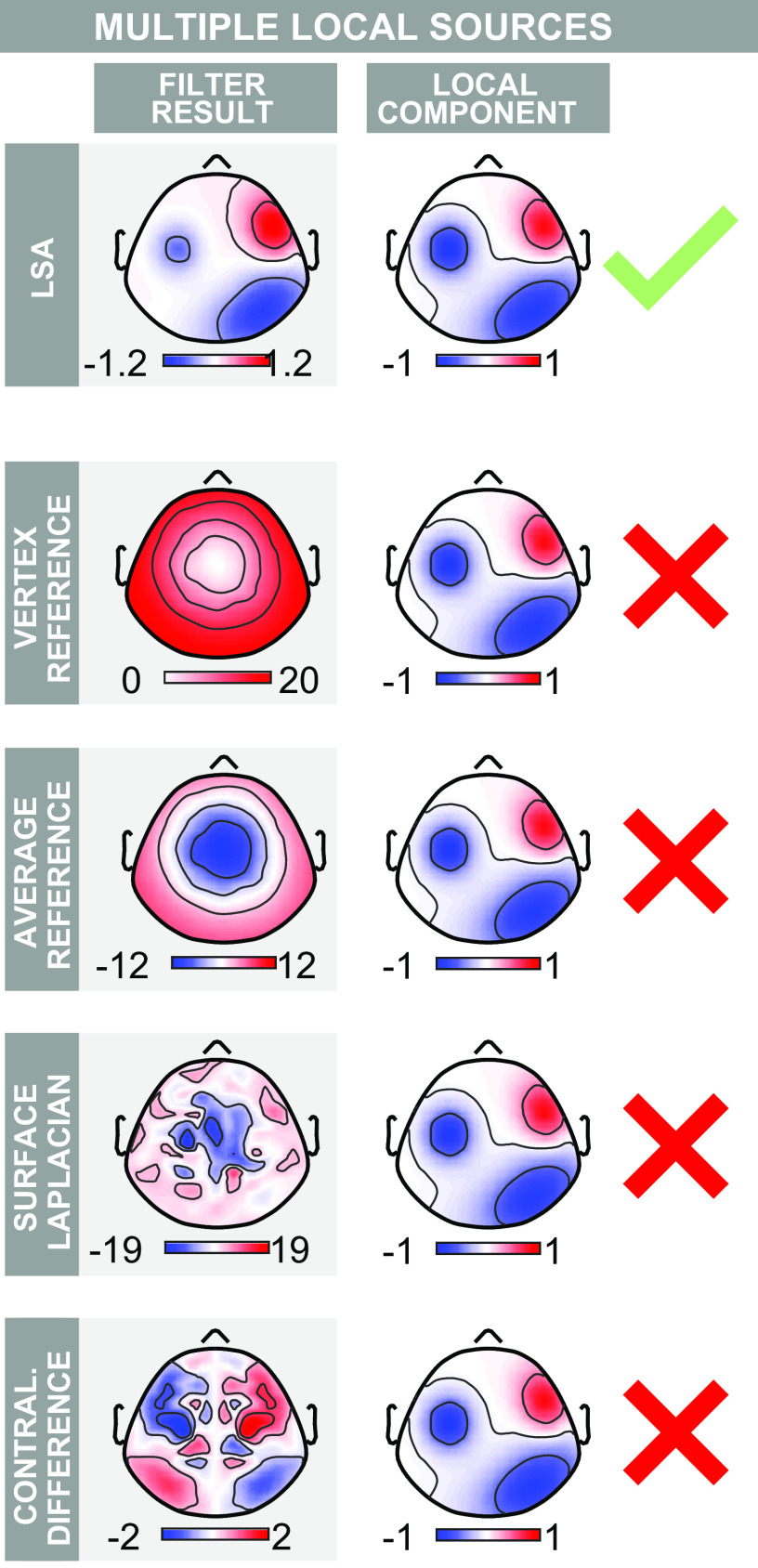
Performance of local spatial analysis (LSA) on simulated event-related potential (ERP) data containing a central widespread component and multiple lateralized local components. Layout is as in Fig. 2 but two local components have been added. LSA successfully returns a scalp topography virtually identical to that of the multiple original local components. Commonly used stationary filters fail to highlight the local components. The contralateral difference merges two of the local sources, because it returns spurious components on opposite hemispheres. Color axes represent voltage (µV).

**Figure 5.**
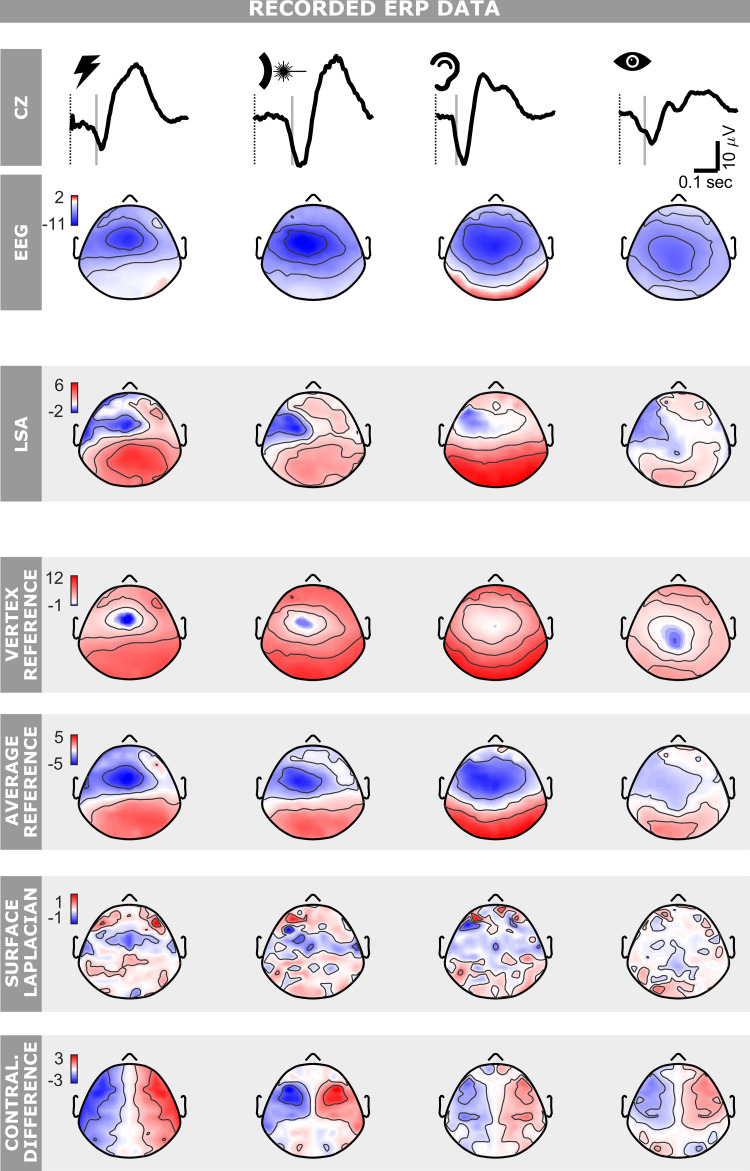
Performance of local spatial analysis (LSA) on recorded EEG data. Event-related potentials (ERPs) were elicited by fast-rising nonnociceptive (electric) somatosensory, nociceptive (laser) somatosensory, auditory, and visual stimuli delivered at long and variable interstimulus interval of 5–10 s (columns from *left* to *right*). The first two rows illustrate the ERP waveform at Cz and the scalp topography at the latency of the grey vertical line, i.e., 30 ms before the peak of the N2 negative wave (see methods). The third row shows the output of LSA at the same latency. The remaining rows show the output, again at the same latency, of commonly used stationary filters: vertex reference, average reference, surface Laplacian, and contralateral difference. Note how the local component is best highlighted by LSA. Color axes represent voltage (µV).

Next, we repeated all the above analyses, but adding two additional local sources. The results from the stationary filters were qualitatively similar; VR and AR failed to highlight local activity, SL returned spatially noisy maps in which individual components could not be distinguished, and CD created components with spurious symmetrical images. Overall, PCA and ICA performed better than the static filters, but were inconsistent upon repeated simulation, and highly susceptible to noise. LSA, on the other hand, consistently returned each of the three local components (Fig. 4, Supplemental Fig. S5).

### Location-Dependent Activity in the Somatosensory, Auditory, and Visual ERPs

To investigate whether the LSA filter can highlight local activity in real EEG data, we applied it to ERPs elicited by stimuli belonging to different sensory modalities, delivered to or near the participants’ right hands.

#### Somatosensory ERPs.

We began our analysis by investigating the somatosensory N1 (sN1), an ERP component contralateral to the stimulated hand. This component is observed both in response to nociceptive and nonnociceptive somatosensory stimulation. The sN1 is an ideal test-bench for our filter because it overlaps with the larger centrally distributed somatosensory N2 (sN2) (37–39). However, the sN1 is distinct from the sN2. First, the sN1 is maximal over the central electrodes contralateral to the stimulated hand, whereas the sN2 is symmetrical and maximal at the scalp vertex (37). Second, the sN1 peaks ∼30 ms earlier than the sN2 (39). Finally, the sN1 provides information about the activity of the ascending somatosensory pathways complementary to that of the sN2 (21, 34, 40–43).

Importantly, the sN1 was already visible in the spatially unfiltered EEG as a negativity in the hemisphere contralateral to the hand of stimulation, at ∼100 ms and 160 ms poststimulus following nonnociceptive and nociceptive stimulation, respectively. However, the sN1 spatially overlapped the centrally distributed sN2 for both stimulus types (Fig. 5, left two columns). We applied LSA to adaptively remove the sN2 component, captured maximally at Cz, from all other electrodes. The workflow detailing how LSA was used on the somatosensory ERP is shown in Fig. 3. For nonnociceptive somatosensory stimulation, LSA revealed a negative component with a minimum of -2.5 ± 0.9 µV at electrode AF7 (means ± standard error; one-tailed *t* test, *P* = 0.004). For nociceptive somatosensory stimulation, LSA revealed a negative component with a minimum of −2.2 ± 0.5 µV at electrode FCC3H (means ± standard error; one-tailed *t* test, *P* = 0.001). Latency, amplitude, and topography of the extracted components were consistent with previous reports (37, 39, 44).

We also applied the four previously discussed stationary filters to the same somatosensory ERPs (Fig. 5, left two columns). Both AR and VR failed to highlight any lateralization beyond that already observable in the unprocessed EEG. SL did not highlight any lateralization for nonnociceptive somatosensory stimuli, whereas for laser stimuli it returned a negative minimum at electrode FFC5H (−1.8 ± 0.8 µV/cm^2^, means ± standard error; one-tailed *t* test, *P* = 0.02). However, the SL output topography was noisy and did not reveal any clear component; it also included residual activity from central and ipsilateral regions. CD isolated a localized negativity compatible with the sN1 uniquely for nociceptive stimuli: it returned a negativity of −3.9 ± 1.6 µV (means ± standard error; one-tailed *t* test, *P* = 0.02) at FFC5H, but also a symmetrical and spurious positive activity on the hemisphere ipsilateral to the stimulated hand.

#### Auditory ERPs.

We then investigated the auditory ERPs elicited by short transient tones (see methods). Like the somatosensory ERP, the auditory ERP is also dominated by a widespread symmetrical vertex negativity, peaking at ∼100 ms (often referred to as N1, but here for symmetry in nomenclature with the somatosensory modality referred to as aN2). This vertex aN2 overlaps with an earlier negative component (which we refer to as aN1). As expected from previous reports (45–50), we observed the aN1 lateralization in the spatially unfiltered EEG as a negativity in the left hemisphere (i.e., contralateral to the stimulated right side), with a peak latency of ∼80 ms poststimulus (Fig. 5, third column).

Compared with the somatosensory response, highlighting the lateralized auditory aN1 was a more challenging test as in our data the aN2 vertex component was broader than the sN2. LSA revealed a negative component with a minimum of −1.3 ± 0.5 µV at FC3 (means ± standard error; one-tailed *t* test, *P* = 0.016). Latency, amplitude, and topography of the extracted component were consistent with previous reports (45, 51). In contrast, none of the applied stationary filters could highlight this lateralized activity (Fig. 5, third column).

#### Visual ERPs.

When applied to the somatosensory and auditory ERPs, LSA returned a negative component contralateral to the stimulated side and peaking ∼30 ms before the overlapping widespread vertex negativity (Fig. 5, first three columns). Given the similarity of these two results, we investigated whether this lateralized component was also present in the ERPs elicited by brief flashes presented in the right visual hemifield (see methods).

The visually evoked negative vertex wave peaked 137 ms poststimulus (Fig. 5, left column, top plot). For this reason, we applied LSA to the ERP response at 107 ms, i.e., 30 ms before the negative vertex peak. At this latency LSA revealed a lateralized negative component with a minimum of −1.6 ± 0.4 µV at electrode FT7 (one-tailed *t* test, *P* = 0.0004; Fig. 5, right-most column). Latency, amplitude and topography of this isolated component were similar to those observed in the early local components of somatosensory and auditory ERPs. Similarly to what we observed in the auditory ERP, none of the stationary methods revealed this lateralized component in the visual ERPs (Fig. 5, right-most column).

#### Somatosensory, auditory, and visual ERPs following left-side stimulation.

One of the analyzed datasets (19) also contained nonnociceptive somatosensory, auditory, and visual stimulation delivered to and near the left hand. The results from the identical analysis described earlier (*Location-Dependent Activity in the Somatosensory, Auditory, and Visual ERPs* section), but applied to the left side stimulation are shown in Supplemental Fig. S6. We again found local components contralateral to the stimulated side for both somatosensory and visual stimulation (−2.5 ± 0.7 6 µV at FT8, *P* = 0.002 for electric; −1.1 ± 0.5 µV at FT8, *P* = 0.02 for visual). For auditory stimulation, a localized, negative, contralateral component was visible in the grand average of the LSA, but this result was not statistically robust (−0.9 ± 0.9 µV at F6, *P* = 0.18). In fact, auditory left-side stimulation was one of the few situations where the SL outperformed LSA, showing a localized negative component on the frontal right hemisphere (−1.6 ± 0.7 µV at F4, *P* = 0.02), and a relatively noise-free scalp topography. Notably, a local negativity ipsilateral to stimulation side was present for auditory stimuli (−1.0 ± 0.5 µV at FC3, *P* = 0.04), and a more widespread ipsilateral negativity was present for visual stimuli (−2.0 ± 0.7 µV at FT7, *P* = 0.009).

## DISCUSSION

Here, we describe a new filter for extracting local ERP components masked by large widespread activity (local spatial analysis, LSA). We hope that LSA can serve as an example of a new way of performing EEG analyses: instead of blindly apply a certain spatial filter to the entire ERP time course, filters can be developed ad hoc, to tackle the particular question that researchers face. Specifically, LSA exploits the information stored in the trial-by-trial variability of the ERP response to extract the local activity that, for each electrode and time point, is statistically independent to the widespread activity. Thus, LSA is adaptive both in space and time. Using simulated data, we show that *1*) LSA can extract local ERP components using few tens of trials, even when the signal-to-noise ratio is low. Using real data, we show that *2*) LSA highlights well-known local and small ERP components—the somatosensory (sN1) or the auditory (aN1)—that overlap with strong and widespread scalp negativities. Furthermore, we identified a frontal local component of the visual ERP (vN1) that is reminiscent of the somatosensory and auditory N1s. To the best of our knowledge, this vN1 has not been described before. Importantly, *3*) LSA outperformed all commonly used stationary filters in both recorded and simulated data.

Finally, LSA is considerably easier to use than other popular adaptive filters such as ICA and PCA. We discuss the implications of these findings in the remainder of the DISCUSSION.

### LSA Allows Extracting Local Components in Simulated Data

We applied LSA to simulated EEG data. LSA reliably highlighted the local activity when 40 trials were generated for one single time point (Fig. 2). This result is important for two reasons. First, it shows that LSA can work effectively with the number of trials collected in typical ERP experiments, i.e., between tens and hundreds. Second, it shows that LSA can successfully highlight local activity at single time points (see also the discussion about time-stationarity of conventional adaptive filters in the section *LSA Is Simpler than Conventional Adaptive Spatial EEG Filters*). This last result is not trivial. Indeed ICA, another adaptive filtering technique, failed to highlight the simulated local components on the same simulated data (Supplemental Fig. S3 and S5), although PCA performed somewhat better (Supplemental Fig. S4 and S5).

### LSA Allows Extracting Local Components from the Somatosensory, Auditory, and Visual ERPs

We also applied LSA to real ERPs elicited by stimuli belonging to different sensory modalities: auditory, visual, and somatosensory (both nociceptive and nonnociceptive). These scalp ERPs are functionally heterogeneous and reflect the activity of distinct cortical generators overlapping in time and space (1). When elicited by isolated and intense fast-rising stimuli (as in the case of the datasets analyzed in this study), large and widespread scalp potentials dominate over small and local potentials (19, 52). Therefore, these ERPs provide an appropriate test-bench for an algorithm that aims to isolate small-amplitude local components embedded within large-amplitude widespread activity.

By removing widespread scalp activities, LSA effectively isolated local centrofrontal negativities contralateral to the stimulated side, in all sensory modalities. These local negativities have been previously described in the somatosensory and auditory domains. Specifically, in somatosensory ERPs elicited by isolated and intense transient stimuli (37, 39), there is converging evidence in both human and rodents that this early negative component is generated in the primary sensorimotor cortex contralateral to the stimulated hand or forepaw (53). In auditory ERPs, such a lateralized negativity is maximal over frontal-central electrodes contralateral to the stimulated auditory hemifield (45–50), and its generators have been suggested to be located in the superior temporal gyrus (Brodmann’s Area 22) (54). To the best of our knowledge, a negative component of this kind has not been previously described in visual ERPs. A lateralized subcomponent of the visual N2 has been shown to be contralateral to the visual hemifield of stimulation (55); however, this component has a more occipital distribution than the lateralized component extracted with our method.

These early local components extracted by LSA in all sensory modalities share several features: they are all negative, contralateral to the stimulated side, centrofrontally distributed, and of similar amplitude. Finally, in all modalities, they are maximal approximately 30 ms before the peak latency of the subsequent negative vertex wave (Fig. 5).

Even though a detailed discussion of the origin of this component is beyond the scope of this article, on the basis of these similarities it is tempting to speculate that there is a common neural mechanism responsible for producing at least part of this negativity in all modalities. Using the component disclosed by LSA as input data for subsequent source analysis could possibly help clarify this issue. Sensorimotor areas could play a role in the generation of this lateralized component. Indeed, we have recently shown that saliency-evoked EEG responses are tightly coupled with a modulation of the motor output (56). Importantly, the correlation strength is maximal for the centrofrontal EEG activity contralateral to the stimulus. Such a sensorimotor explanation might also fit with the fact that when stimuli were presented near the left hand, both ipsilateral and contralateral local activity were present. This observation is consistent with the well-known asymmetry of cortical motor function: processes related to the nondominant hand are often more bilaterally distributed than processes related to the dominant hand (57–59). It is also possible that this local contralateral component arises due to some features of the tested task, given that participants were counting the stimuli (60). Clearly, the relationship between the negativities highlighted by LSA and motor activity deserves further investigation.

### LSA Outperforms Commonly-Used Stationary Spatial Filters

We compared the performance of LSA in isolating local EEG activity to that of four commonly used stationary filters. LSA outperformed all stationary techniques considered: stationary methods could not retrieve the local components in simulated data (Fig. 2) nor highlight the well-known lateralized sN1 and aN1 in recorded EEG data (Fig. 5). These results provide evidence that stationary techniques can be unsuited for extracting meaningful spatial features in ERP responses, because ERP responses are intrinsically nonstationary. We now critically review each stationary filter and compare it to LSA.

We compared the result of our filter to that obtained using the vertex reference (VR) for two reasons. First, VR is widely used in asymmetry research for highlighting EEG lateralizations (2–8). Second, given that in both simulated and recorded data we used LSA to remove Cz activity from that of all other electrodes, VR and LSA performed identical operations, except for one aspect. VR used a single fixed weight for all electrodes, whereas LSA adaptively estimated the weight for each electrode and subject from the data. Our results clearly show that this difference is crucial: unlike LSA, VR failed to highlight any lateralized component (Fig. 5). Our findings support previous conclusions discouraging the use of the vertex reference in EEG analysis (6, 14, 61).

The average reference (AR) is probably the most commonly used stationary spatial EEG filter (1). Its success stems from the fact that the scalp topographies obtained with this technique appear to be more spatially localized when widespread activity is present. However, this filter does not alter the spatial structure of the data. It simply shifts the EEG signal at all sites by an identical amount. As detailed elsewhere (1), when only the scalp region of the head is sampled (as in most EEG experiments), the average across electrodes is dominated by the electrode where the widespread activity is maximal. In this case the change in color scale of the scalp maps, from a widespread positive or negative voltage to a mixture of positive and negative voltage within a given timeframe, is intuitively but erroneously interpreted as highlighting local components. In reality, there is no change whatsoever in the spatial relationships between the EEG at different sites. This was also the case in our data (Fig. 5). For this reason, as already suggested by others, we propose that AR should only be used blindly when the entire head of the subject (i.e., including face, chin, jaws. and neck) is covered with a high number of electrodes, as only in this case the average of all electrodes can be considered approximately neutral (10).

The surface Laplacian (SL) collectively denotes a group of mathematical operations that attempt to transform the recorded EEG into values of the radial current flow at the scalp, by estimating the second order spatial derivative of the EEG signal (10, 11). Several implementations of this technique exist. The core principle, however, is the same for all implementations and consists in computing local weighted differences for estimating the spatial derivatives (11). Both in simulated and recorded data, SL returned maps that were spatially noisy and hard to interpret in terms of underlying local components (Figs. 2 and 5). This is no surprise given that from the signal processing point-of-view the SL is a high-pass spatial filter (10); it dampens widespread effects and highlights local spatial variability. Although this might seem similar to achieving the objective of highlighting local activity, the issue is that SL highlights “any” type of spatial variability, independently of whether this is introduced by brain activity or by differences in electrode conductance or electrical noise.

The contralateral difference (CD) is the standard procedure for extracting the so-called Lateralized Readiness Potential (LRP) (1, 30) and for highlighting differences in EEG activity between the two hemispheres (54, 62). In our data, CD did not highlight the local lateralized activity that was instead revealed by LSA in auditory and visual ERPs (Fig. 5). Furthermore, our simulations revealed that the use of CD poses three main issues. First, CD cannot highlight local components close to the midline. Second, more worryingly, CD is susceptible to artifacts due to even small shifts in cap placement along the mediolateral axis. These artifacts can become an issue when trying to identify local sources close to the midline such as the N1 wave in somatosensory ERPs elicited by foot stimulation (39, 44, 63). In this case, if the cap is slightly displaced in the mediolateral direction, CD can return a spurious local source close to the midline, which can be misinterpreted as a sensorimotor source (Supplemental Fig. S2). Finally, unless the EEG data are entirely symmetrical, CD always produces a spurious, symmetrical activity of opposite polarity along the mediolateral axis.

### LSA Is Simpler than Conventional Adaptive Spatial EEG Filters

We did not perform an exhaustive comparison between the results of LSA and those of other adaptive filters using the recorded data (although we did such a direct comparison using simulated data, see the *Simulations* section under results and Fig. 2). In principle, there is no reason to assume that similar results could not be obtained using other conventional adaptive filters. However, even assuming that these filters would yield the same results, their use is more burdensome than LSA, to the point of being impractical. To illustrate this, let’s consider the differences between LSA and ICA. We chose ICA among the popular adaptive filters for two reasons. First, ICA is the most commonly used adaptive spatial filter; it has now become standard for removing physiological artifacts such as blinking and heartbeat (1, 29, 64) and it can be used to separate ERP components (19–21). Second, similarly to LSA, ICA separates components on the basis of the assumption that the voltages produced by different sources should be, to some extent, statistically independent. Although we do not discuss PCA and beamforming in similar detail for the sake of brevity, we note that several of the issues highlighted here for ICA also apply to these methods.

We start by noting that understanding the way LSA works is much easier than understanding how ICA works. While LSA uses a simple linear regression, ICA uses a combination of techniques that require advanced statistical knowledge such as whitening, random weights initialization, and maximization of measures of statistical dependency (e.g., kurtosis, negentropy, Kullback–Leibler divergence) (31). Many researchers, therefore, are likely to apply ICA without a full understanding of its underlying principles. This difference is important: when users understand the analytics behind a certain method, they are less likely to misuse it or misinterpret its results.

To demonstrate the extent to which using ICA can be more burdensome than LSA, we compare the procedure for isolating the somatosensory N1 with LSA— described in this work—with that for performing the same task using ICA—outlined in a previous study from our group (37). To highlight the lateralized N1 components with LSA only two steps were needed: *1*) selecting the midline electrode where the widespread N2 is strongest in the grand average EEG and *2*) running the algorithm to automatically remove the activity of the chosen electrode for each subject through trial-wise linear regression (Fig. 3). In contrast, the ICA workflow required considerably more complicated steps: *step 1*) running ICA to extract the independent components (ICs); *step 2*) categorizing the ICs into stimulus-related and nonstimulus-related components using a Z-score comparison against the prestimulus interval; *step 3*) selecting the stimulus-related ICs with a peak latency of the N2 between 175 ms and 275 ms; *step 4*) visually inspecting these ICs to identify those with a scalp topography centrally distributed and maximal at the vertex, and finally, *step 5*) removing the ICs identified at *step 4*. Note that these steps must be performed separately for each subject and that some of them require time-consuming visual inspections of single-subject ICs. The sequence of steps highlights two shortcomings of ICA. First, the advantage of using a “blind” (i.e., assumption-less) technique such as ICA is usually lost during IC categorization, which requires subjective prior knowledge and assumptions. Supplemental Fig. S7 illustrates that if such knowledge is not used, neither ICA nor PCA, which also relies on prior knowledge when used across subjects, can successfully identify local components in the real data. Second, the IC categorization is complex and time-consuming. It is not surprising that researchers often refrain from using ICA and opt instead for the less-effective, but more immediate, stationary methods.

There is one additional issue that makes the use of ICA more impractical than LSA for obtaining the results described here. LSA adapts the filter weights at each time-point using only the information stored in the trial-by-trial variability at that specific time point. LSA is thus adaptive both in time and space. ICA, instead, pools together EEG values from the whole ERP time course and from all trials, to create a large statistical dataset for estimating the large number of ICs. Crucially, by mixing EEG data collected at different time points, ICA is not sensitive to small transient EEG changes, which are an object of interest of ERP analysis. In other words, ICA is adaptive in space but not in time. Possibly for this reason, in two previous studies (19, 21) from our group using the same data, ICA failed to highlight the lateralized auditory activity. To reproduce with ICA the results yielded by LSA (Fig. 5), we would need to apply ICA only to the time points of the response at which we expect to observe the lateralized components. However, properly categorizing the ICs extracted using such a small amount of data would be extremely hard if not impossible, given that these ICs would include a large amount of noise and minimal time information. Given that PCA and beamforming are also not adaptive in time, none of the most commonly used adaptive filters can be used in the simple way that LSA allows. The ability of LSA to adapt to even small and short-lasting EEG changes is one of its most important advantages. The implications of this advantage are discussed in the next section.

### A New Approach to Spatial EEG Filtering

An open issue in EEG analysis is how to establish best practices to spatially filter the data (65). As few of the available stationary or adaptive spatial filters have been developed with a specific question in mind, the standard approach has been to compare the effect of each filter on a number of test-bench datasets, to identify the filters providing the best results (12, 65). The issue with this approach is that it tries to match generic tools to specific problems.

In this work, we propose an alternative approach that consists of exploiting the cues visible in the unfiltered EEG, and use them to build filtering tools that work ad hoc for specific situations. This approach, therefore, shapes the tool around the problem, preventing the situation where filters are used on data in which the meaning of their output can be misinterpreted. For example, LSA allows exploiting the presence of a large widespread component in the unfiltered EEG and, thus, to develop a filtering strategy for testing the possible presence of masked underlying local activity. Although LSA is by no means a perfect tool—for example it is linear, whereas the mapping between sources and scalp potentials might not be—we hope that the kind of approach it exemplifies will become more popular. Importantly, this approach needs to be applicable only in the specific time points where the assumptions hold, and therefore needs to be adaptive in time. For example, one should not use LSA just anywhere on the ERP time course, but only in those windows containing a strong widespread activity in the unfiltered signal. Besides the large vertex waves dominating the responses reported in this work (21), other examples are the large ERPs recorded during motor (the lateralized readiness potential) (66), executive control (the error related negativity) (67) or language tasks (the N400) (68).

In this work, we have demonstrated that building spatial EEG filters that are adaptive in time as well as in space is possible not only in theory but also in practice, even using a dataset with the small number of trials collected in typical EEG experiments. Although we are aware that the approach described here cannot be extended to the entirety of problems in EEG, we believe that this new strategy can be used for creating a new class of time-adaptive spatial EEG filters to address different issues in ERP analyses.

## GRANTS

This work is supported by the Wellcome Trust Strategic Award (COLL JLARAXR) and by the ERC Consolidator Grant (PAINSTRAT) awarded to G. Iannetti.

## DISCLOSURES

No conflicts of interest, financial or otherwise, are declared by the authors.

## AUTHOR CONTRIBUTIONS

R.J.B. and C.M. conceived and designed research; R.J.B. and C.M. performed experiments; R.J.B., C.M., and G.N. analyzed data; R.J.B., C.M., and G.N. interpreted results of experiments; R.J.B. and C.M. prepared figures; C.M. and G.D.I. drafted manuscript; R.J.B., G.N., and G.D.I. edited and revised manuscript; R.J.B., C.M., G.N., and G.D.I. approved final version of manuscript.
